# Three-Dimensional Live Imaging of Bovine Preimplantation Embryos: A New Method for IVF Embryo Evaluation

**DOI:** 10.3389/fvets.2021.639249

**Published:** 2021-04-26

**Authors:** Yasumitsu Masuda, Ryo Hasebe, Yasushi Kuromi, Masayoshi Kobayashi, Kanako Urataki, Mitsugu Hishinuma, Tetsuya Ohbayashi, Ryo Nishimura

**Affiliations:** ^1^Department of Animal Science, Tottori Livestock Research Center, Tottori, Japan; ^2^SCREEN Holdings Co., Ltd., Kyoto, Japan; ^3^Laboratory of Theriogenology, Joint Department of Veterinary Medicine, Faculty of Agriculture, Tottori University, Tottori, Japan; ^4^Organization for Research Initiative and Promotion, Tottori University, Tottori, Japan

**Keywords:** optical coherence tomography, embryo, 3D image, embryo transfer, quantification of embryo structures

## Abstract

Conception rates for transferred bovine embryos are lower than those for artificial insemination. Embryo transfer (ET) is widely used in cattle but many of the transferred embryos fail to develop, thus, a more effective method for selecting bovine embryos suitable for ET is required. To evaluate the developmental potential of bovine preimplantation embryos (2-cell stage embryos and blastocysts), we have used the non-invasive method of optical coherence tomography (OCT) to obtain live images. The images were used to evaluate 22 parameters of blastocysts, such as the volume of the inner cell mass and the thicknesses of the trophectoderm (TE). Bovine embryos were obtained by *in vitro* fertilization (IVF) of the cumulus-oocyte complexes aspirated by ovum pick-up from Japanese Black cattle. The quality of the blastocysts was examined under an inverted microscope and all were confirmed to be Code1 according to the International Embryo Transfer Society standards for embryo evaluation. The OCT images of embryos were taken at the 2-cell and blastocyst stages prior to the transfer. In OCT, the embryos were irradiated with near-infrared light for a few minutes to capture three-dimensional images. Nuclei of the 2-cell stage embryos were clearly observed by OCT, and polynuclear cells at the 2-cell stage were also clearly found. With OCT, we were able to observe embryos at the blastocyst stage and evaluate their parameters. The conception rate following OCT (15/30; 50%) is typical for ETs and no newborn calves showed neonatal overgrowth or died, indicating that the OCT did not adversely affect the ET. A principal components analysis was unable to identify the parameters associated with successful pregnancy, while by using hierarchical clustering analysis, TE volume has been suggested to be one of the parameters for the evaluation of bovine embryo. The present results show that OCT imaging can be used to investigate time-dependent changes of IVF embryos. With further improvements, it should be useful for selecting high-quality embryos for transfer.

## Introduction

Bovine embryo transfer (ET) has been widely used to produce calf in combination with other reproductive technologies, such as *in vitro* fertilization (IVF). However, the conception rate of ET using IVF embryos (30–40%) is lower than that of using embryos produced *in vivo* (around 50%) (1–4). Embryos for transfer are usually selected by observation under a conventional optical microscope at the time of transfer, and embryo quality is subjectively assigned as one of the codes according to the International Embryo Technology Society (IETS) standards for embryo evaluation (5, 6).

In human artificial reproductive technology (ART), embryos are evaluated based on the Veeck and Gardner classification (7, 8) and time-lapse cinematography (TLC) with a visible light microscope, which has recently become a popular technology. Morphokinetic parameters, such as the number of pronuclei or nuclei, timing of cleavage, and the number of blastomeres, are used as potential indicators that may improve the success of ART (9). Furthermore, ART success rate has been improved by comprehensive chromosomal screening using techniques such as array comparative genomic hybridization, quantitative single nucleotide polymorphism arrays, and next-generation sequencing (10, 11). To evaluate *in vitro* developed bovine embryos, TLC has been used to determine the time of the first cleavage and the subsequent number of blastomeres, and the number of blastomeres at the onset of the lag-phase (4, 12–14). However, so far, live bovine embryos have not been evaluated based on their three-dimensional (3D) structure.

A morphological grading system in human ART was first described by Gardner and Schoolcraft (15). According to this system, three parameters (degree of blastocoel expansion, size and compactness of ICM, and the cohesiveness and number of TE) are graded. Based on these criteria, an additional consensus on embryo assessment was agreed including new references for each parameter (16, 17). In this consensus, ICM grade is suggested to be more important for determining the implantation potential of a blastocyst. To select the best blastocyst when performing ET on Day 5, several parameters have been suggested to contribute to the implantation potential of blastocyst. Some investigators have shown that the timing of blastocoel development and the grade of expansion are important parameters for implantation (18–20). Other investigators have suggested that the size and shape of ICM are related to implantation (21–23). Either a positive association or no association of TE cells with implantation has been reported (22, 24–26). Morphological grading, while common for human blastocysts, is difficult for bovine blastocyst because of their dark cytoplasm (27, 28).

Optical coherence tomography (OCT) has been developed for non-invasive, cross-sectional imaging in biological systems (29–31), and is presently used in ophthalmology, especially for funduscopic examination of the retina. OCT can be used to measure 3D images with high spatial resolution, because it can scan small biological structures, such as micro vessel structures during *in vitro* angiogenesis (31). Recently, OCT imaging of mouse (32–34) and porcine (34) early-stage embryo has been reported. In the mouse blastocysts, their nucleoli were also clearly visualized by OCT (34). In cattle, blastocysts have been imaged by OCT, and their cytoplasm movements that are potentially associated with viability were monitored, suggesting that OCT can be used for the measurement of the damage after cryopreservation (35). However, the quantification of the structures of bovine blastocysts for evaluating embryo quality has not been reported. We have recently developed a technique for the 3D imaging of bovine blastocysts and used it to evaluate 22 parameters including the volumes of the ICM, TE, zona pellucida (ZP) and blastocoel of an embryo (36). Here, we used this technique to compare the characteristics of embryos that did or did not develop to term in order to identify the parameters associated with successful ET. Furthermore, in the blastomere observation, the shape, size, cytoplasm color, even distribution of cytoplasm, and number of nucleus have been suggested to be related to the developmental potential of bovine embryos (4, 12–14). Because bovine and porcine early embryos contain much more lipid than human or mouse embryos, pronucleus formation in early embryos cannot be confirmed under a microscope, which made it difficult to evaluate their quality (27, 28, 37, 38). Thus, we have also tried to obtain 3D images of early-stage bovine embryos.

## Materials and Methods

### Ethics Statement

Animal handling and experimental procedures were carried out following the Guidelines for Proper Conduct of Animal Experiments by the Science Council of Japan (http://www.scj.go.jp/ja/info/kohyo/pdf/kohyo-20-k16-2e.pdf).

### Production of Embryos Derived From Oocytes Collected by Ovum-Pick-Up (OPU) and *in vitro* Maturation (IVM)

As described previously (39), cumulus-oocyte complexes (COCs) were collected from Japanese Black cows (*n* = 12; 110.4 ± 34.3-month-old) by OPU using an ultrasound scanner (HS-2100; Honda Electronics, Toyohashi, Japan) and a 7.5-MHz convex array transducer (HCV-4710MV; Honda Electronics) with a 17-gauge stainless steel needle guide (242 COCs, in total). Follicles >2 mm in diameter were aspirated with a vacuum through a disposable aspiration needle (COVA Needle; Misawa Medical, Tokyo, Japan). The aspiration rate was 14 mL/min and the vacuum pressure was 100 mmHg. The IVM medium was 25 mM HEPES-buffered TCM199 (M199; Gibco, Paisley, Scotland, UK), supplemented with 10% newborn calf serum (NCS; 16010159, Gibco) and 0.01 AU/mL of follicle-stimulating hormone from porcine pituitary (Antorin-R10; Kyoritsu Seiyaku, Tokyo, Japan). COCs with two or more granulosa layers were washed three times with IVM medium. Recovered COCs were cultured in 4-well dishes (Non-Treated Multidishes; Nalge Nunc International, Roskilde, Denmark) in 600 μL of IVM medium, covered with mineral oil (M8414; Sigma-Aldrich, St. Louis, MO, USA), and incubated for 22 h at 38.5°C in 5% CO_2_, 5% O_2_, and 90% N_2_ in humidified air. All cultures were maintained under these conditions.

### IVF

Frozen semen of Japanese Black bulls stored in straws was thawed in water (37°C, 40 sec), and then centrifuged twice in IVF100 (Research Institute for the Functional Peptides, Yamagata, Japan; 600 × *g*, 5 min). After centrifugation, spermatozoa were removed from the pellet, and added to IVF100 to obtain a suspension with a final sperm concentration of 1.0 × 10^7^/mL. This suspension served as the IVF medium. After 22 h of IVM, the COCs were washed twice with IVF100. Up to 20 COCs were incubated in 100 μL droplets of IVF medium in 35 mm dishes (Falcon 351008; Corning, NY, USA) for 6 h.

### *In vitro* Culture (IVC)

After insemination, oocytes were completely denuded from the cumulus cells and spermatozoa by pipetting with a glass pipette in IVC medium: potassium simplex optimized medium with amino acid (KSOMaa Evolve Bovine; Zenith Biotech, Bangkok, Thailand) supplemented with 5% NCS and 0.6 mg/mL of L-carnitine (C0158, Sigma-Aldrich). Subsequently, presumptive zygotes were washed three times with IVC medium and cultured in 100 μL droplets of IVC medium for 48 h. Each droplet contained approximately 20 presumptive zygotes. Average value of cleavage rate was 74.0% (179/242). At 48 h post-insemination (hpi), embryos with more than four cells were transferred from the 35 mm dishes to well-of-the-well (WOW) dishes (LinKID micro25, Dai Nippon Printing Co., Ltd., Tokyo, Japan) as described (12). WOW dishes, which have 25 microwells (5 columns × 5 rows) in a circular wall in the center of a 35 mm dish, can culture up to 25 embryos each with a single drop of medium and track individual embryos throughout the culture. IVC medium and mineral oil were pre-cultured for at least 12 h in glass bottles separately at 38.5°C in 5% CO_2_, 5% O_2_, and 90% N_2_ in humidified air, and the pre-cultured IVC medium (100 μL) was placed within the circular wall and covered with the pre-cultured mineral oil. At 168 to 180 hpi, embryos that had developed to or beyond the blastocyst stage were observed under an inverted microscope. Finally, 123 embryos had developed to the blastocyst stage (50.8%), and 80 blastocysts had been cryopreserved.

### OCT Observations

**Figure 1:**
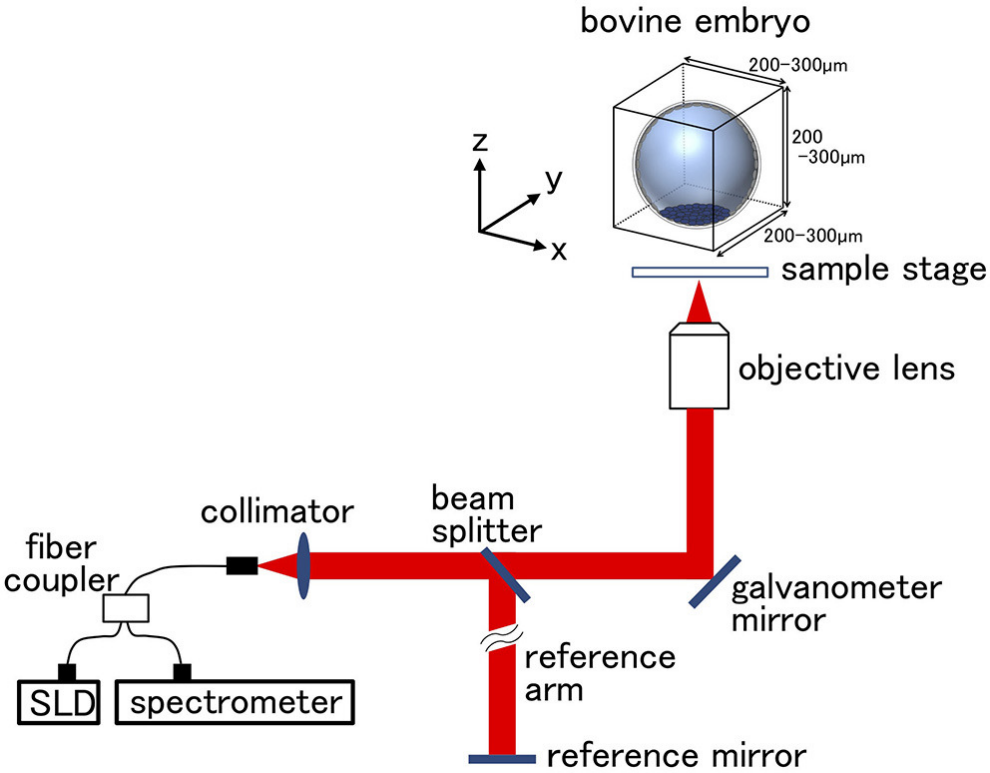
Optical coherence tomography (OCT) setup. The super luminescent diode (SLD) output is coupled into a single mode fiber and split at the fiber coupler into the embryo sample and reference arms. Reflections from the two arms are combined at the coupler and detected by the spectrometer. Scanning scale for the bovine embryo was 200–300 μm in each direction. Longitudinal imaging was performed in the area of bovine embryo.

IVF embryos were cultured for seven days (by this time, they reached the expanded blastocyst stage) and examined under an inverted microscope at 27–31 hpi (at the 2-cell stage; *n* = 15) and at 168 to 180 hpi (at the blastocyst stage; *n* = 30). In blastocysts, only the embryos that were independently classified as Code1 according to the IETS standards by three skilled observers were used. OCT imaging was done as described previously (31, 36). Unstained live embryos were imaged by OCT using the Cell3iMager Estier (SCREEN Holdings Co., Ltd, Kyoto, Japan). The imaging system of Cell3iMager Estier is outlined in Figure 1. The system is equipped with a super luminescent diode (SLD; center wavelength: 890 nm, N.A. = 0.3). The SLD output is coupled to a single-mode optical fiber and split at an optical fiber coupler into the sample and reference arms. The reflections from the two arms are combined at the coupler and detected by the spectrometer. The 3D image data of the blastocysts were constructed from individual 2D ×-z cross-sectional images, which were obtained by a series of longitudinal scans obtained by laterally translating the optical beam position. The data acquisition window was 200–300 × 200–300 × 200–300 μm, and the voxel size was 1 × 1 × 1 μm. The OCT system scans light source positions in the x-axis direction while the shifting scanning line positions on the y-axis to obtain a signal on the x–y plane at a focus position on the z-axis. By repeating this scanning while shifting the z-axis focus positions, 3D images of the embryos were acquired. The lengths of the imaging range on the x-, y-, and z-axis were 300, 300, and 200 μm, respectively. The exposure time was 150 μs, and scanning an entire embryo was completed in a few minutes. OCT provided cross-sectional images with a slice thickness of 1 μm.

### Cryopreservation

As described previously (40), blastocysts imaged by OCT were transferred to a cryoprotective solution [1.8 M of ethylene glycol and 0.1 M of sucrose in Dulbecco's PBS (D-PBS)], which was then placed in a 0.25-mL straw (IMV Technologies, L'Aigle, France) at room temperature. After the blastocysts were equilibrated at room temperature for 15 min, the straws were directly set in a programmable freezer (ET-1N; FUJIHIRA INDUSTRY CO., LTD, Tokyo, Japan) at −7°C where seeding was manually performed. Subsequently, the straws were cooled at a rate of 0.3°C/min to −30°C and then directly transferred to liquid nitrogen for storage until use. The straws were thawed in air for 10 s and then immersed in water (30°C, 20 s) for the ET. Because of the status of our farms, we have chosen a cryopreservation protocol rather than a verification protocol.

### Image Analysis

**Figure 2:**
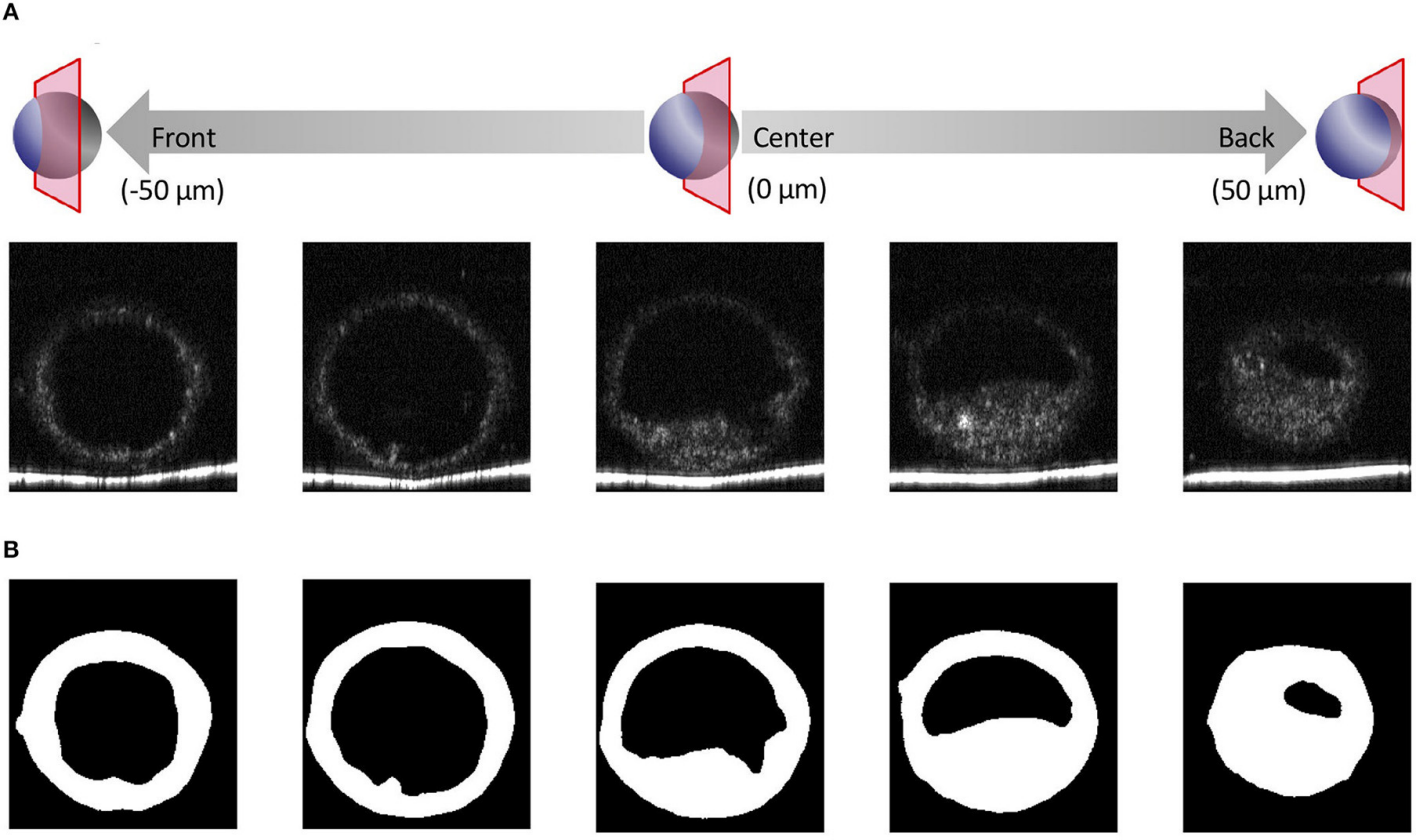
Optical coherence tomography (OCT) images of the bovine embryo and each structure of an embryo. **(A)** Tomographic images obtained by OCT imaging. Panels are images shifted by 25 μm from the center of the embryo. **(B)** Based on the tomographic images, the structure of the embryo is visualized and binarized.

**Figure 3:**
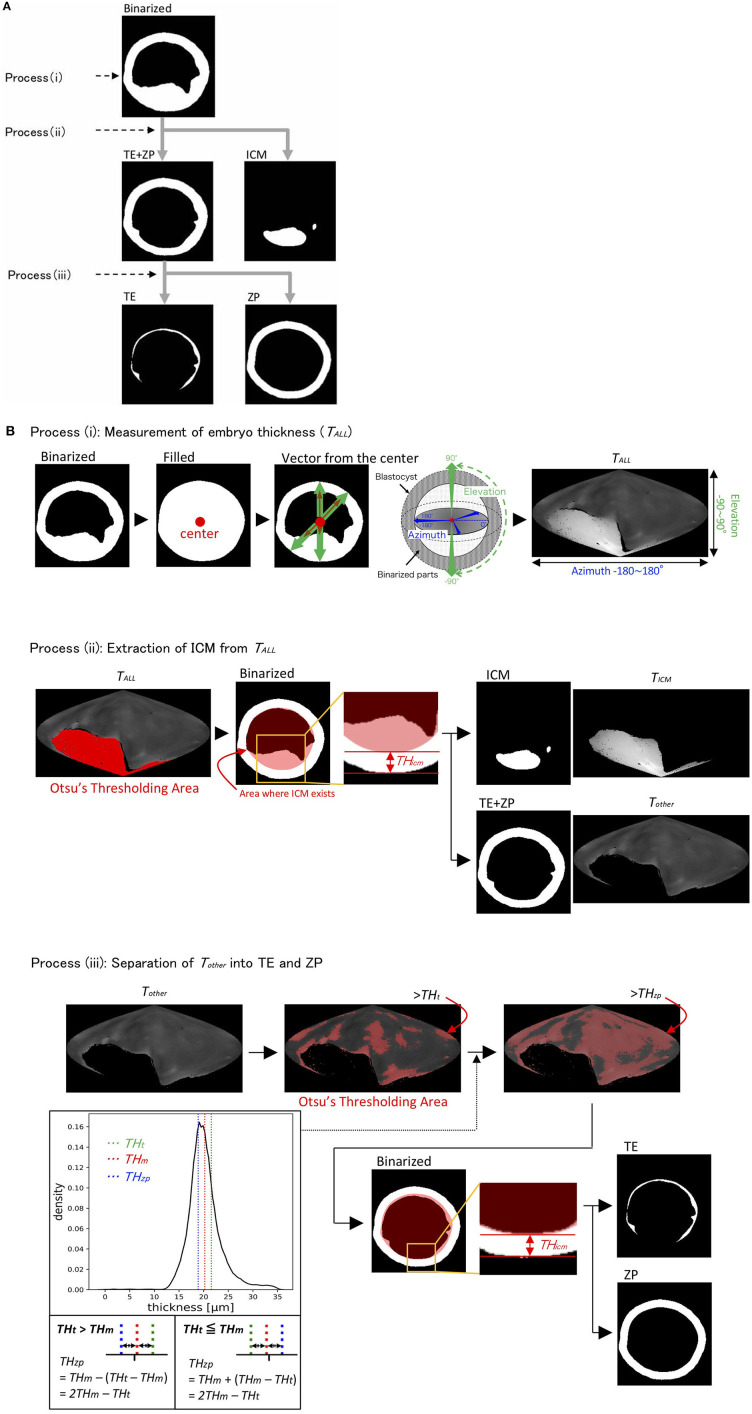
The binarized image was separated into the inner cell mass (ICM), trophectoderm (TE), and zona pellucida (ZP) through processes (i)–(iii) **(A)**. **(B)** Process (i): Thickness of embryo (*T*_*ALL*_) was measured by drawing vectors from the center to the outer and inner surfaces (the outermost of blastocoel) of the embryo. Process (ii): ICM parts were extracted from *T*_*ALL*_ by Otsu's thresholding method (41, 42). Process (iii): The parts, which remained after removing the ICM parts from *T*_*ALL*_, were defined as *T*_*other*_. *T*_*other*_ was separated into TE and ZP by calculating the average thickness of *T*_*other*_ (*TH*_*m*_) and by using Otsu's thresholding method (41, 42). *T*_*All*_, thicknesses of embryo along each vector; *TH*_*icm*_, threshold of ICM; *T*_*ICM*_, thickness of ICM; *T*_*other*_, thickness of the areas other than ICM; *TH*_*t*_, threshold of *T*_*other*_; *TH*_*m*_, average of *THt*; *TH*_*zp*_, thickness of ZP (=2 *TH*_*m*_*-TH*_*t*_).

We recently reported using OCT to image bovine embryos (36). After the 3D images were captured by OCT (Figure 1), the 3D images were analyzed in an automized way (Figures 2, 3). Process (i) The 3D images of bovine embryos were binarized (Figures 2, 3). For each image, vectors were drawn at equal angles in the elevation direction (−90–90°) and the azimuth direction (−180–180°) from the center of the embryo to the outer surface and the inner surface (the outermost of blastocoel). The thicknesses of the embryo along each vector (*T*_*All*_) were then measured (Figure 3). Process (ii) To distinguish the ICM from *T*_*All*_, an appropriate threshold (*TH*_*icm*_; the cut-off value for distinguishing ICM from *T*_*All*_) was set by Otsu's method (41, 42) (Figure 3). The parts where the thickness from the outer edge of the embryo region was lower than *TH*_*icm*_ were excluded from the binarized images. The largest object among the remaining objects after the exclusion was defined as ICM. *T*_*ALL*_ was separated into the thickness information of the area corresponding to ICM (*T*_*ICM*_) and the thickness information areas other than ICM (*T*_*other*_). Process (iii) Based on the *T*_*other*_ value, the average thickness (*TH*_*m*_) was evaluated, and the threshold (*TH*_*t*_) was set by Otsu's method (41, 42) (Figure 3). The threshold (*TH*_*zp*_) that separates TE and ZP was set by *TH*_*m*_–*(TH*_*t*_*-TH*_*m*_*)* (in case of *TH*_*t*_ > *TH*_*m*_) or by *TH*_*m*_ + *(TH*_*m*_*-TH*_*t*_*)* (in case of *TH*_*m*_ ≧ *TH*_*t*_). The region where the thickness of *T*_*other*_ was lower than *TH*_*zp*_ was defined as ZP, and the remaining region after removing *TH*_*zp*_ from *T*_*other*_ was defined as TE. The unfulfilled region, surrounded by the embryo parts, such as ICM, ZP, and TE, in the binarized image was defined as the blastocoel. The volumes of the defined ICM, TE, ZP, and blastocoel were evaluated. The means, medians, standard deviations, minimum, maximum, and range of the thickness of ICM were evaluated from *T*_*ICM*_. The thickness of TE was evaluated from the thickness information, which was obtained by subtracting *TH*_*zp*_ from *T*_*other*_. Summary statistics related to the thickness of ZP was set by *TH*_*zp*_.

### ET and Pregnancy Diagnosis

The OCT-imaged embryos were transferred to 30 recipient cows [47.5 ± 25.5-month-old Holstein (18 lactating cows) and Japanese Black cows (10 cows; two cows received ET twice)] from March 2018 to February 2019 at the farms in Tottori prefecture, Japan. The cows were clinically normal with body condition scores (BCS) between 2.75 and 3.0 (BCS scale goes from 1 to 5 with 0.25 increments). Before ET, recipients were estrus-synchronized by the administration of a CIDR device (CIDR 1900; Zoetis Japan, Tokyo, Japan) for 9 days and a treatment with cloprostenol (Dalmazin 150 μg [i.m.]; Kyoritsu Seiyaku Corporation, Tokyo, Japan) 2 days before the CIDR removal. Estrus of recipient cows was monitored, and embryos were transferred seven days after estrus with the confirmation of the presence of corpus luteum (CL; diameter ≧20 mm). The recipients were examined for pregnancy 23 days after ET using ultrasonography (HS101V; Honda Electronics). Pregnancy was confirmed in nine Holstein cows and six Japanese Black cows by observation of a CL ≧ 20 mm in diameter and an embryo with a detectable heartbeat in the intraluminal uterine fluid and an embryonic membrane. Ages of pregnant and non-pregnant cows were 42.3 ± 19.9 and 52.7 ± 29.5-months-old, respectively. Cows with BCS of 2.75 and 3.0 were included equally in both pregnant and non-pregnant cows.

### Data Analysis

Hierarchical clustering analysis was performed using 13 blastocoel-related and ZP-related parameters of bovine blastocysts. Metrics and linkage criteria for hierarchical clustering were Pearson's correlation and unweighted average linkage. The data was normalized so that the *average* = *0* and *SD* = *1* for each of the parameter. Hierarchical clustering analysis was performed using the SciPy (ver.1.3.0) package in Python (ver.3.6.5). Statistical significance was analyzed using the Mann-Whitney *U* test. A value of *p* < 0.05 was considered statistically significant. Mann-Whitney *U* test was performed using the SciPy (ver.1.3.0) package in Python (ver.3.6.5). Principal component analysis (PCA) was performed using 22 parameters of bovine blastocysts. The data was normalized so that the *average* = *0* and *SD* = *1* for each of the parameter. PCA was performed using the scikit-learn (ver.0.23.2) package in Python (ver.3.6.5).

## Results

### 3D Imaging of Bovine IVF Embryos at the 2-Cell Stage

**Figure 4:**
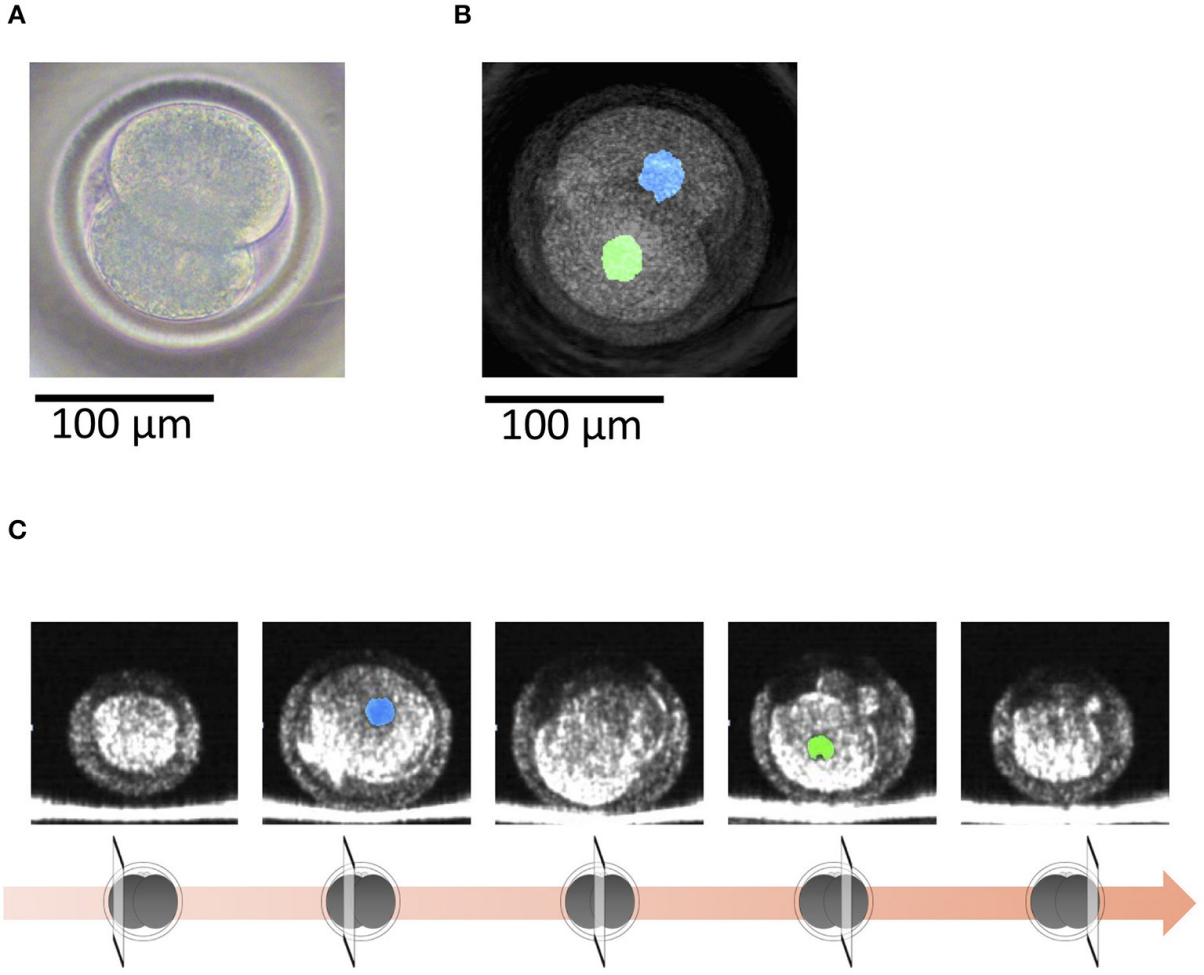
**(A)** A bovine 2-cell embryo imaged by a microscope. **(B)** Optical coherence tomography (OCT) images of the bovine 2-cell embryo with visualization of the nucleus (blue and green) of the blastomere. **(C)** Tomographic images obtained by OCT imaging. Panels are images shifted by 25 μm from the center of the embryo with visualization of the nucleus (blue and green) of the blastomere.

**Figure 5:**
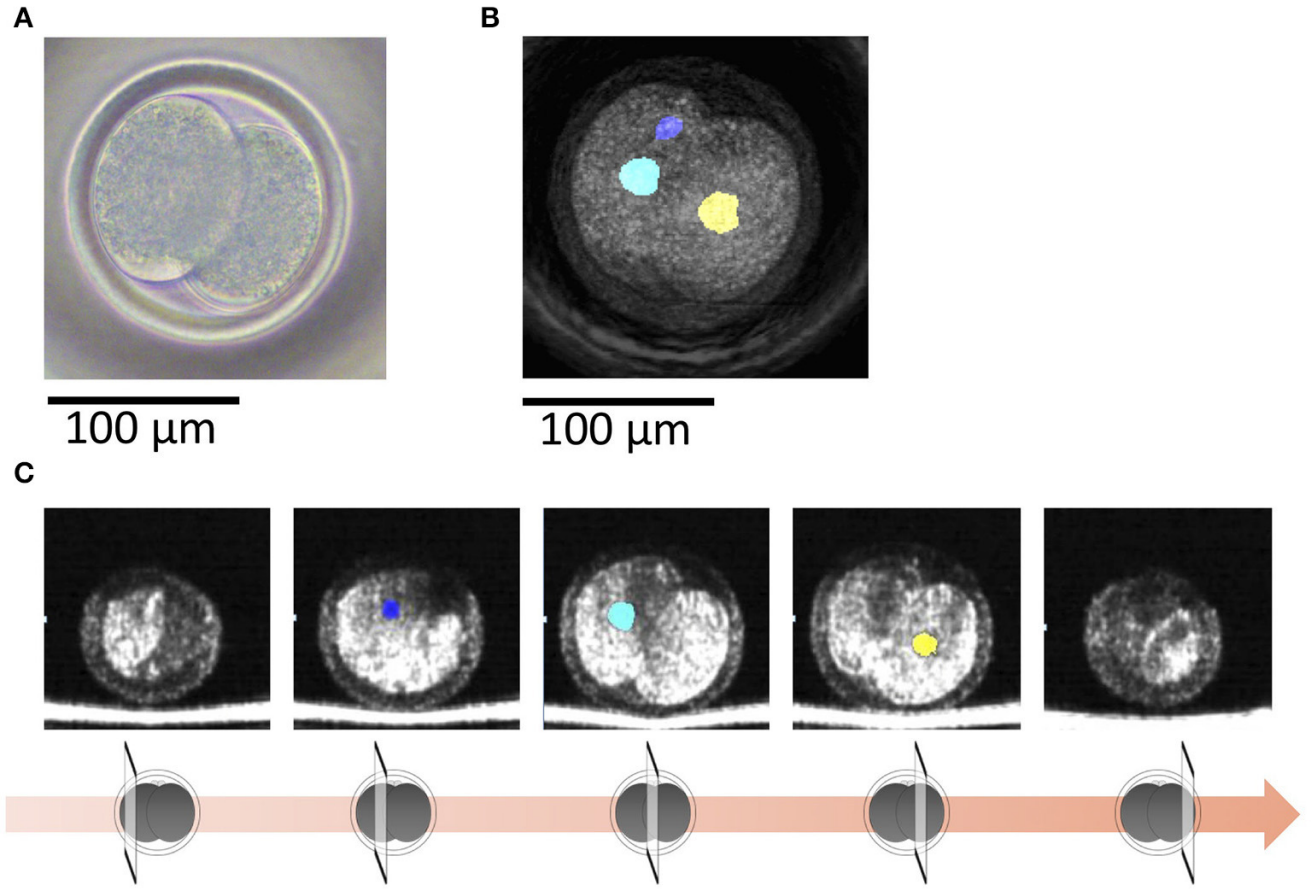
**(A)** A bovine 2-cell embryo including a binuclear blastomere imaged by a microscope. **(B)** Optical coherence tomography (OCT) images of the bovine 2-cell embryo including a binuclear blastomere with visualization of the nucleus (blue, green, and yellow) of the blastomere. Blue and green nucleus are in a blastomere. **(C)** Tomographic images obtained by OCT imaging. Panels are images shifted by 25 μm from the center of the embryo with visualization of the nucleus (blue, green, and yellow) of the blastomere.

With OCT, it was possible to non-invasively obtain live 3D images of a 2-cell embryo. OCT images also showed the nuclei (Figures 4B,C), which made it easy to find binuclear blastomeres (blue and green in Figures 5B,C). Of the 15 embryos examined by OCT, two were binuclear and were not used for transfer.

### Quantification of Bovine Embryo Morphology at the Blastocyst Stage and ET Success Rate

**Figure 6:**
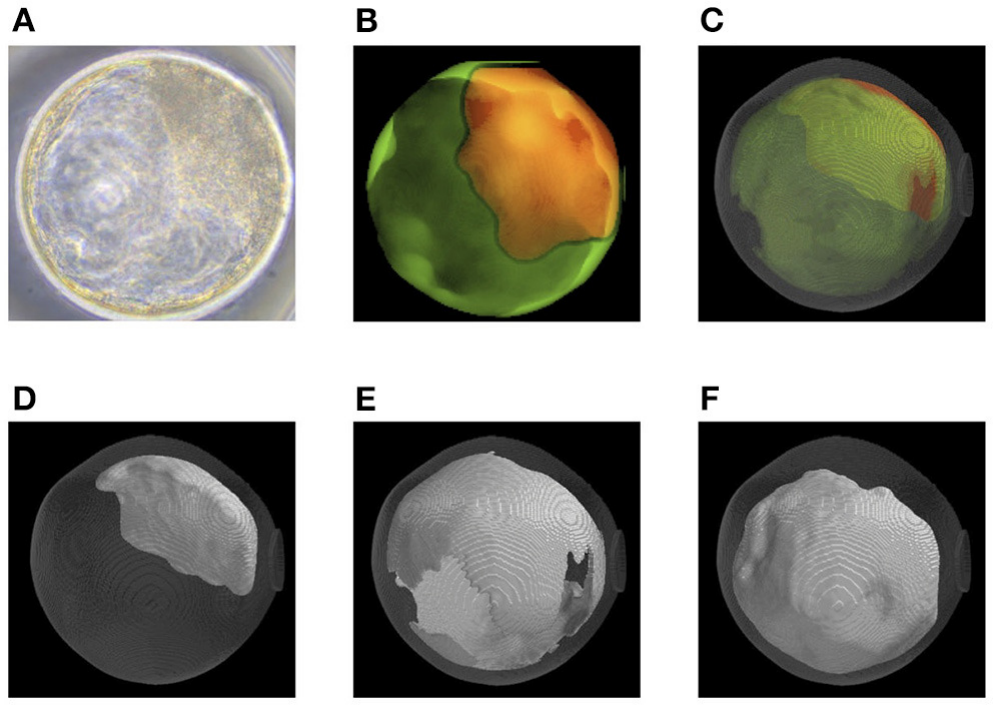
Representative optical coherence tomography (OCT) images of a transferred bovine embryo that resulted in pregnancy. **(A)** A representative transferred embryo, determined as Code 1 according to the IETS standards, imaged by a microscope. **(B)** Sum of all pixel values in z-stack images of trophectoderm (TE; bright green) and inner cell mass (ICM; orange) part was extracted from the tomographic image and synthesized 2D image. **(C)** A 3D-visualization of structures of the embryo, including ICM (orange), TE (bright green), zona pellucida (ZP; gray), and blastocoel. **(D–F)** A 3D visualization of each structure of the embryo: ICM **(D)**, TE **(E)**, and blastocoel **(F)**. Colors in **(B–F)** are graphically added. See also Supplementary Movies 1–4 for the 3D images of **(C–F)**, respectively.

**Figure 7:**
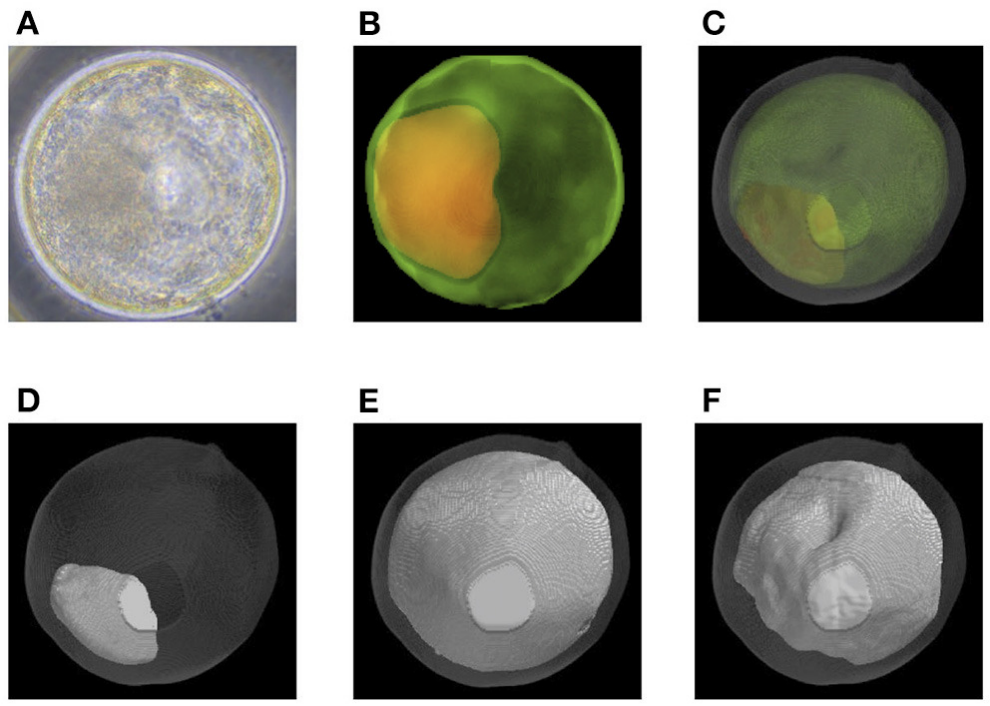
Representative optical coherence tomography (OCT) images of a transferred bovine embryo that resulted in non-pregnancy. **(A)** A representative transferred embryo, determined as Code 1 according to the IETS standards, imaged by a microscope. **(B)** Sum of all pixel values in z-stack images of the trophectoderm (TE; bright green) and inner cell mass (ICM; orange) part was extracted from the tomographic image and synthesized 2D image. **(C)** A 3D-visualization of structures of the embryo, including ICM (orange), TE (bright green), zona pellucida (ZP; gray), and blastocoel. **(D–F)** A 3D visualization of each structure of the embryo: ICM **(D)**, TE **(E)**, and blastocoel **(F)**. Colors in **(B–F)** are graphically added. See also Supplementary Movies 5–8 for the 3D images of **(C–F)**, respectively.

Before transfer, we obtained images of an embryo with a microscope (Figures 6A, 7A) and with OCT. OCT images of the trophectoderm (TE) and inner cell mass (ICM) of representative embryos resulting in pregnancy (P embryos) and non-pregnancy (NP embryos) are shown in Figures 6B, 7B, respectively. In these figures, TE and ICM were artificially colored green and orange, respectively. The structure of a whole embryo, including ICM (orange), TE (green), and zona pellucida (ZP; gray) were also 3D-visualized (Figures 6C, 7C; Supplementary Movies 1, 5). Each part of the embryo, the ICM (Figures 6D, 7D; Supplementary Movies 2, 6), TE (Figures 6E, 7E; Supplementary Movies 3, 7), and blastocoel (Figures 6F, 7F; Supplementary Movies 4, 8), was also 3D-visualized individually.

**Figure 8:**
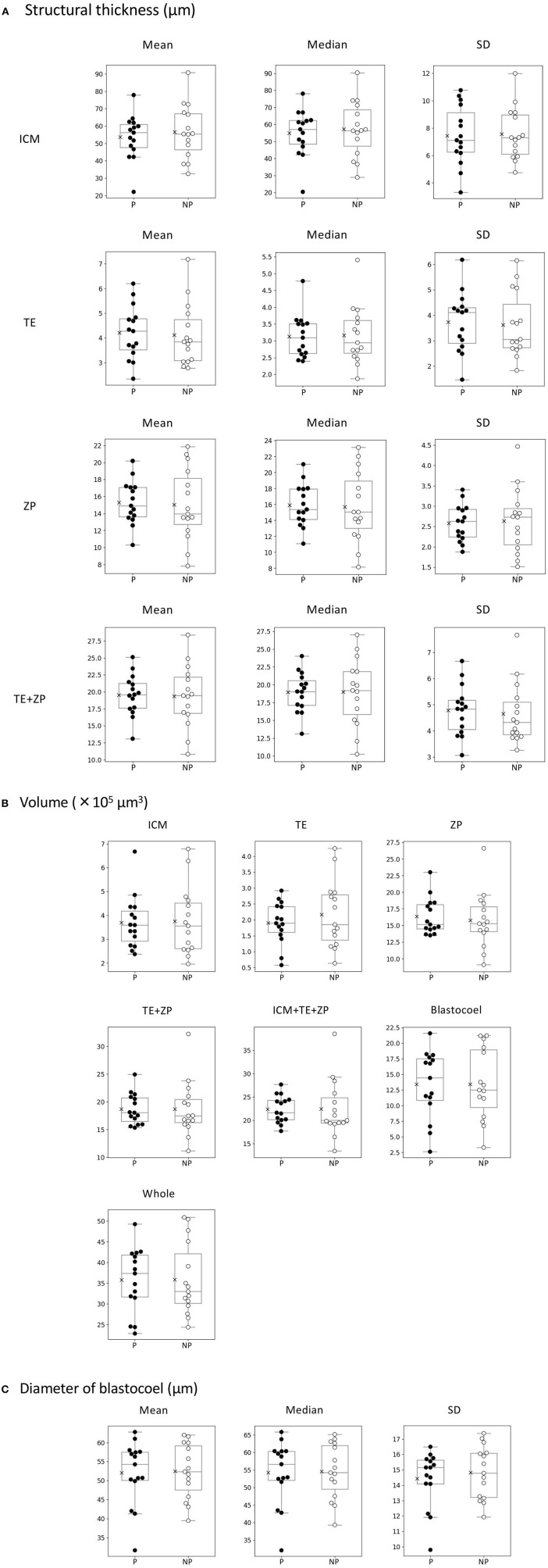
Comparison of 22 morphological parameters of bovine blastocysts between pregnancy (P: *n* = 15) and non-pregnancy (NP: *n* = 15). **(A)** Parameters related to structural thickness including mean, median, and standard deviation (SD) of inner cell mass (ICM), trophectoderm (TE), zona pellucida (ZP), and TE + ZP. Parameters related to the volume of each part of blastocyst (ICM, TE, ZP, TE + ZP, ICM + TE + ZP, blastocoel, and whole embryo). **(C)** Parameters related toblastocoel diameter (mean, median, and SD). Statistical significance was analyzed using the Mann-Whitney *U* test. A value of *p* < 0.05 was considered statistically significant.

Twenty-two parameters were measured for each of the 30 embryos (Table 1). For the embryos that were subjected to ET (*n* = 30), the average thicknesses of ICM, TE, ZP, and TE + ZP were 55.1 ± 14.1, 4.2 ± 1.2, 15.2 ± 3.4, and 19.5 ± 3.8 μm, respectively (mean ± SD). The average volumes of ICM, TE, ZP, TE + ZP, ICM + TE + ZP, blastocoel, and whole embryo were 3.7 ± 1.3, 2.0 ± 0.9, 16.1 ± 3.5, 18.7 ± 4.0, 22.4 ± 4.7, 13.4 ± 5.5, and 35.8 ± 8.1 × 10^5^ μm^3^, respectively (mean ± SD), and the blastocoel diameters were 52.3 ± 7.7 μm (mean ± SD). Fifteen of the 30 recipients became pregnant. None of the parameters were significantly different between the embryos that did (P) or did not (NP) lead to pregnancy (Table 1; Figure 8). Twelve of the 15 pregnant cows gave birth (six males and six females), and the remaining three cows experienced a late embryonic death. Newborn calves had typical weights (male: 40.2 kg; female: 35.5 kg, in average), and did not show any congenital defects, neonatal overgrowth, and death.

**Table 1:** Quantification of 22 parameters in bovine embryo (Pregnancy: *n* = 15, Non-pregnancy: *n* = 15).

		**Pregnancy**			**Non-pregnancy**			***p-*value**	***p* < 0.05**
		**Median**	**Min**	**Max**	**Median**	**Min**	**Max**		
**Structural thickness (μm)**									
	Mean	56.2	22.1	77.8	55.4	32.5	90.7	0.7	n.s.
ICM	Median	57.1	20.4	78.1	56.4	28.9	90.4	0.8	n.s.
	SD	7.1	3.3	10.8	7.3	4.8	12.0	1.0	n.s.
	Mean	4.3	2.3	6.2	3.8	2.8	7.2	0.8	n.s.
TE	Median	3.1	2.4	4.8	2.9	1.9	5.4	0.9	n.s.
	SD	4.1	1.5	6.2	3.1	1.8	6.1	0.8	n.s.
	Mean	14.9	10.3	20.2	13.9	7.8	21.9	1.0	n.s.
ZP	Median	15.4	11.1	21.0	15.0	8.1	23.1	1.0	n.s.
	SD	2.6	1.9	3.4	2.7	1.5	4.5	0.9	n.s.
	Mean	19.6	13.1	25.1	19.4	10.8	28.4	1.0	n.s.
TE + ZP	Median	19.1	13.1	24.0	19.2	10.2	27.0	0.8	n.s.
	SD	4.8	3.1	6.7	4.3	3.3	7.7	0.4	n.s.
**Volume (×10^5^ μm^3^)**									
ICM		3.6	2.4	6.7	3.6	2.0	6.8	1.0	n.s.
TE		1.9	0.6	2.9	1.9	0.6	4.2	1.0	n.s.
ZP		15.1	13.5	23.0	15.3	9.1	26.6	0.5	n.s.
TE+ZP		18.1	15.4	25.0	17.5	11.2	32.3	0.5	n.s.
ICM+TE+ZP		21.7	17.7	27.7	20.0	13.4	38.5	0.4	n.s.
Blastocoel		14.4	2.6	21.6	12.5	3.3	21.2	0.9	n.s.
Whole embryo		37.4	22.9	49.3	33.0	24.4	50.9	0.6	n.s.
**Diameter of blastocoel (μm)**									
Mean		54.3	31.6	62.8	52.3	39.5	62.0	0.8	n.s.
Median		56.7	32.1	65.8	54.2	39.3	65.2	0.8	n.s.
SD		15.1	9.8	16.5	14.8	11.9	17.4	0.8	n.s.

*ICM, inner cell mass; TE, trophectoderm; ZP, zona pellucida*.

### PCA of 22 Morphological Parameters of Bovine Blastocysts

**Figure 9:**
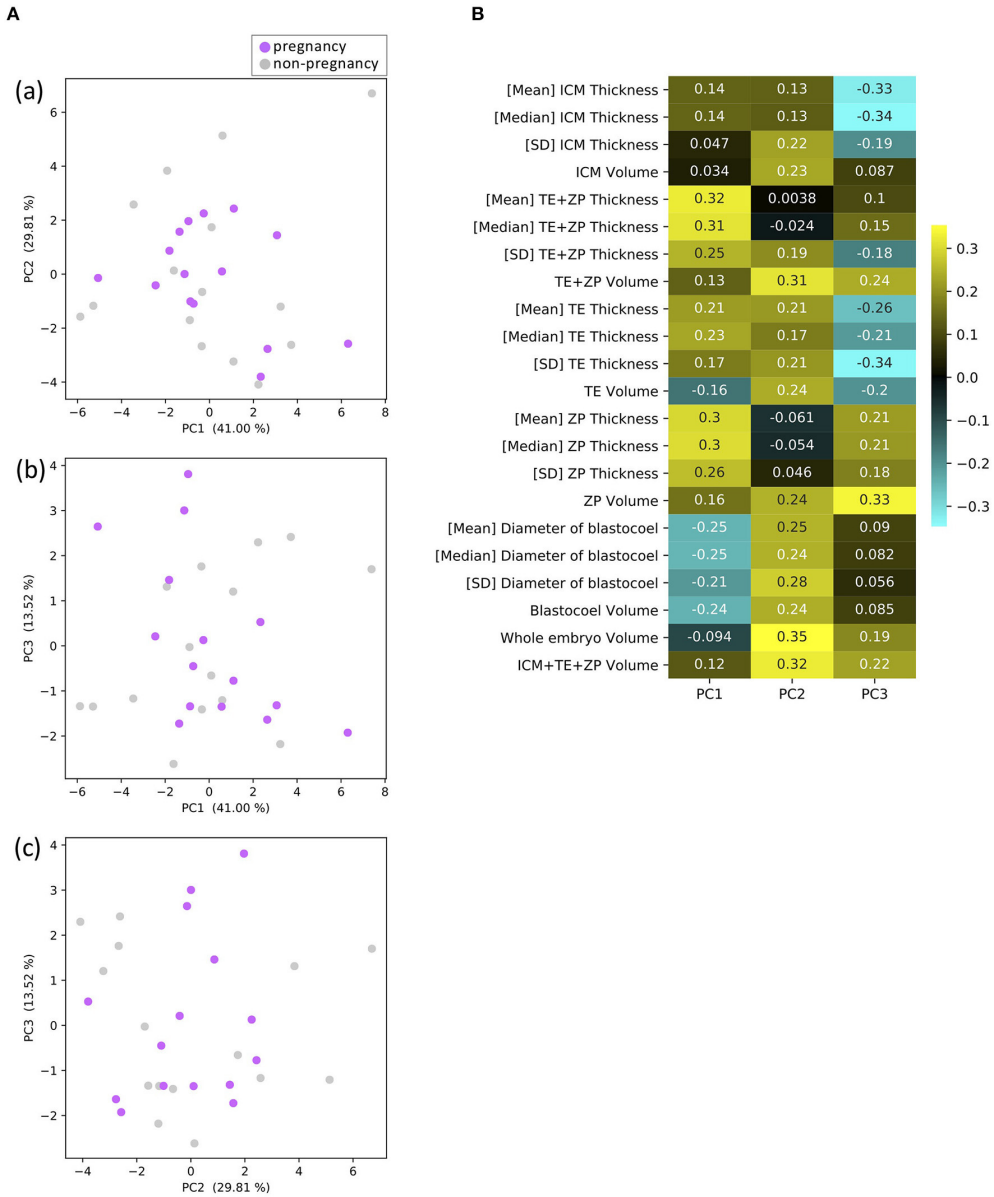
Results of principal component analysis (PCA) based on the 22 morphological parameters of bovine blastocysts (pregnancy: *n* = 15, non-pregnancy: *n* = 15). **(A)** Two-dimensional PCA plots [**(a)** PC1–PC2, **(b)** PC1–PC3, **(c)** PC3–PC2] profiled based on the morphological parameters evaluated from the OCT-scanned 3D images of bovine blastocysts. Each purple and gray dot represents blastocyst resulting in pregnancy and non-pregnancy, respectively. **(B)** Eigenvectors for PC1, 2, and 3.

A PCA identified three principal components, PC1, PC2, and PC3. PC1 was related to the thickness of ZP and TE, which accounted for 41.00% of the variance; PC2 was related to the volumes of parts, which accounted for 29.81% of the variance; and PC3 was related to the thickness of ICM and TE, which accounted for 13.52% of the variance (Figure 9B). Figure 9A shows plots of PC1 vs. PC2 (a), PC1 vs. PC3 (b), and PC3 vs. PC2 (c). None of the plots clearly separated the P and NP embryos.

### Hierarchical Clustering Analysis of the Morphological Parameters of Bovine Blastocysts

**Figure 10:**
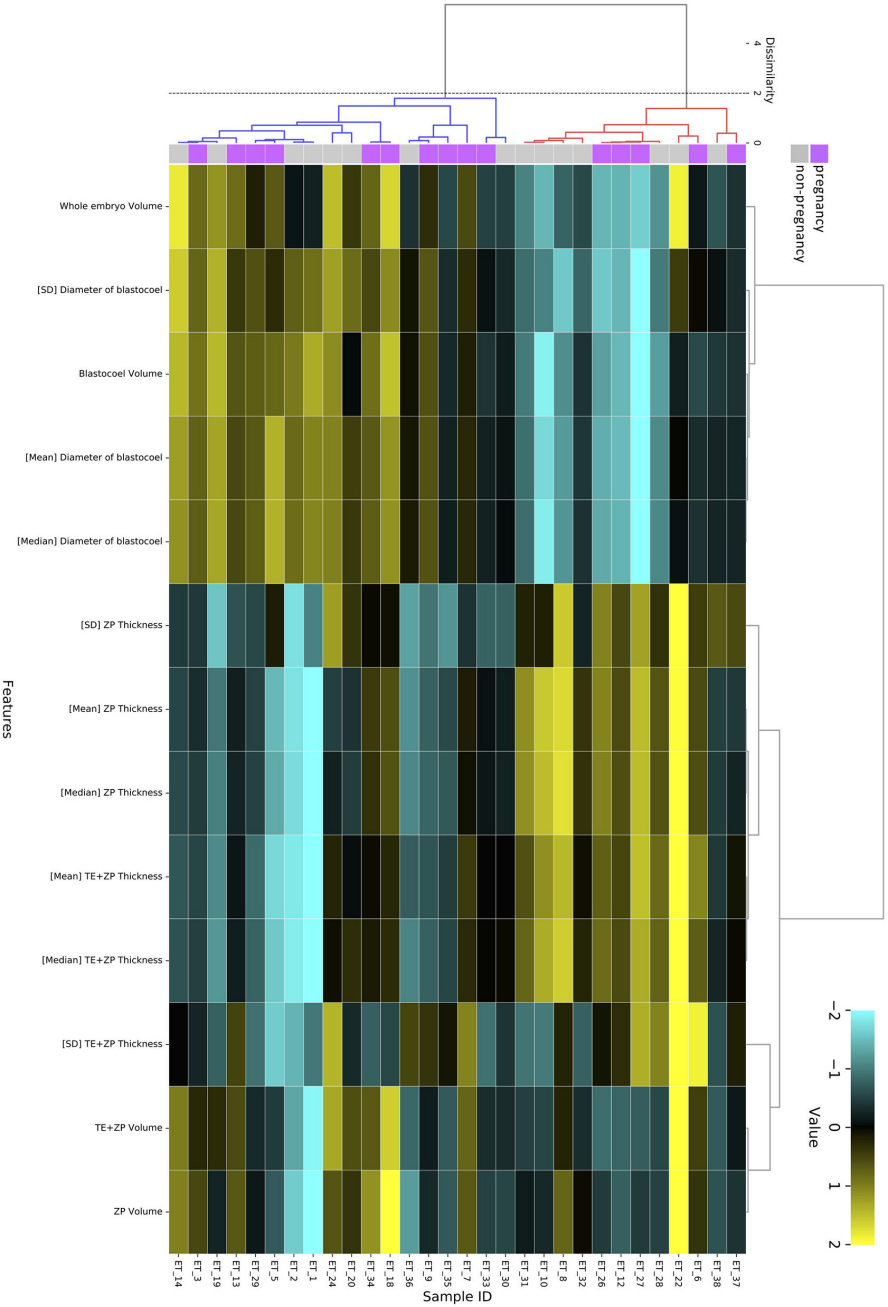
Hierarchical clustering analysis performed using 13 blastocoel-related and ZP-related parameters obtained from 30 blastocysts applied for transfer [pregnancy (purple): *n* = 15, non-pregnancy (gray): *n* = 15]. Embryos displaying similar patterns for these 13 parameters were grouped together on closely connected branches of the dendrogram with the same color. The color map indicates normalized values that were based on the average value of each parameter (*average* = *0* and *SD* = *1*). Yellow represents a high value; black represents approximately an equal to average value; and sky blue represents a low value. Metrics and linkage criteria for hierarchical clustering were Pearson's correlation and unweighted average linkage. Two clusters marked with red and blue were obtained with a threshold of *Dissimilarity* = *2.0*.

**Figure 11:**
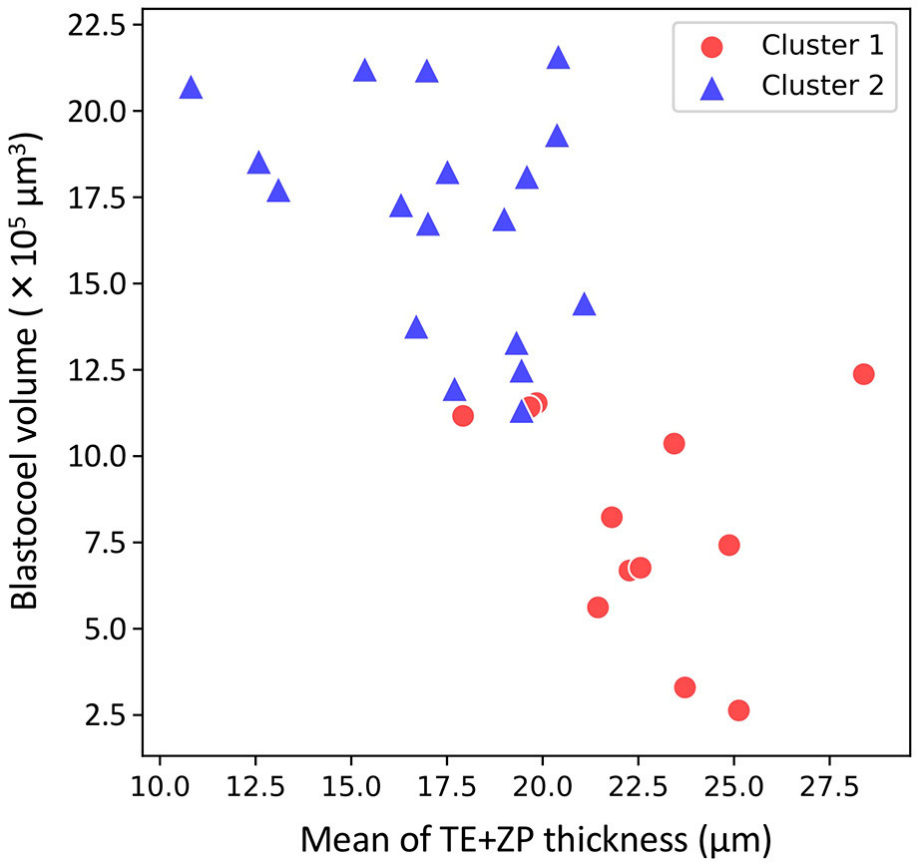
Two-dimensional plots profiled based on the mean of trophectoderm (TE) + zona pellucida (ZP) thickness and blastocoel volume evaluated from the optical coherence tomography (OCT)-scanned 3D images of bovine blastocysts (Cluster 1: red circle, Cluster 2: blue triangle; pregnancy: *n* = 15, non-pregnancy: *n* = 15).

**Figure 12:**
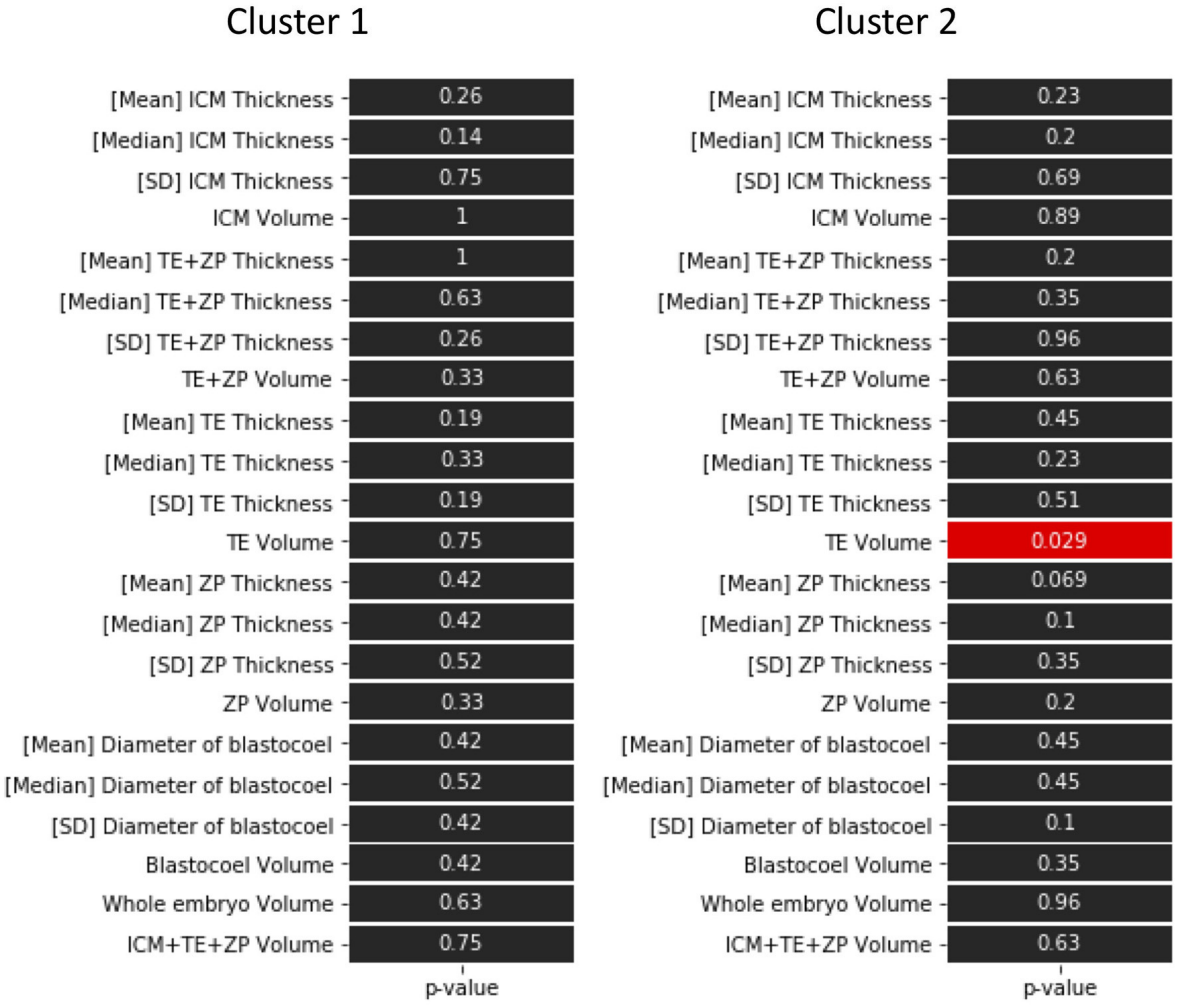
*P*-values in the comparison of 22 parameters evaluated from the optical coherence tomography (OCT)-scanned 3D images of bovine blastocysts between pregnancy and non-pregnancy in each cluster (pregnancy: *n* = 15, non-pregnancy: *n* = 15). Statistical significance was analyzed using the Mann-Whitney *U* test. A value of *p* < 0.05 was considered statistically significant.

**Figure 13:**
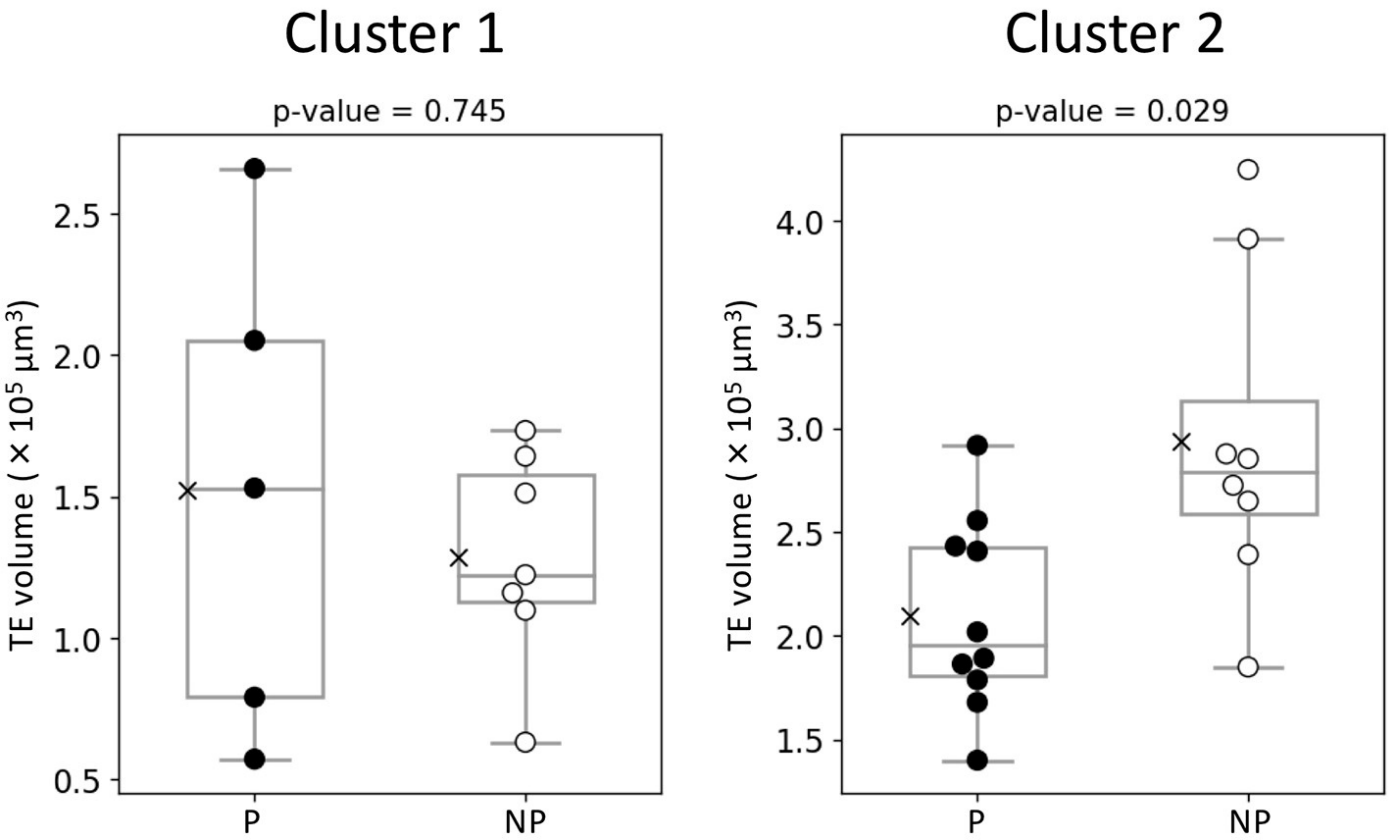
Comparison of TE volume evaluated from the optical coherence tomography (OCT)-scanned 3D images of bovine blastocysts between pregnancy (P) and non-pregnancy (NP) in each cluster (pregnancy: *n* = 15, non-pregnancy: *n* = 15). Statistical significance was analyzed using the Mann-Whitney *U* test. A value of *p* < 0.05 was considered statistically significant.

The hierarchical clustering analysis based on the blastocoel-related and ZP-related parameters (Figure 10) showed two clusters with a threshold of *Dissimilarity* = *2.0*, and these clusters were also found separated into low (Cluster 1) and high (Cluster 2) blastocoel volume clusters on blastocoel volume-TE + ZP thickness plane (Figure 11). In Cluster 1, no significant difference was found in any of the 22 parameters between the P and NP embryos (Figure 12), while in Cluster 2, TE volume was significantly lower in the P embryos (*p* < 0.05) (Figure 12, 13). No difference between the P and NP embryos in any of the 22 parameters was common to both clusters (Figures 12, 13).

## Discussion

The present study describes the 3D images of bovine embryos at the 2-cell and blastocyst stages obtained by OCT. Blastomere nuclei at the 2-cell stage were also clearly visualized by the same system. At the blastocyst stage, 22 morphological parameters were evaluated based on the 3D OCT images. The transfer of 30 bovine embryos after being imaged by OCT resulted in 15 pregnancies (pregnancy rate: 50%) and 12 births (birth rate: 40%), which was typical for the ET attempts (1–4). Bovine blastocysts appeared healthy after a long-term (over 18 h) capture by OCT for monitoring their micro-scale movements (35). These results indicate that OCT can be used to evaluate embryos before ET. As described previously, OCT can capture the inside structure of mammalian embryos (32–36). In addition, the present study has made it possible to quantify several parts of bovine blastocysts including its inside structure, such as the blastocoel, which could not be visualized by conventional microscopy.

A PCA of the measured parameters was unable to find the critical parameters associated with pregnancy. To find the critical parameters for pregnancy, a greater number of transfers of OCT-imaged embryos is needed. As bovine embryos expand into blastocysts, the thickness of the ZP decreases (5). In addition, we detected the blastocoel-related parameters (volume and diameter), which were originally quantified in our recent study by OCT (36). Thus, we conducted a hierarchical clustering analysis based on the blastocoel-related and ZP-related parameters. The hierarchical clustering analysis (Figure 10) shows two clusters with a threshold of *Dissimilarity* = *2.0*, and these clusters were also found separated into two clusters with low (Cluster 1) or high (Cluster 2) blastocoel volumes on a blastocoel volume-TE + ZP thickness plane. While no difference common to both clusters were found in any of the 22 parameters between the P and NP embryos, TE volume in Cluster 2 was significantly lower in the P embryos than in the NP embryos. In Cluster 2, blastocoel volumes were relatively high and the TE + ZP thicknesses were relatively low (Figure 11), suggesting that embryos in Cluster 2 were well-expanded. These results imply that a low TE volume could be one of the parameters for selecting embryo for ET especially in well-expanded blastocysts. However, since in Cluster 1, there was no significant difference in TE volume between the P and NP embryos, more precise methods for the quantification of TE-related parameters and/or a combination of parameters based on the increased number of OCT images are needed to find the critical parameters for the evaluation of bovine embryos for ET. Furthermore, embryo evaluation is more effective if the OCT measurements are done in parallel with time lapse imaging to evaluate other developmental landmarks, such as the timing of embryo cleavage, timing of each developmental stage, and evaluating cell number.

The present study imaged blastomere nuclei at the 2-cell stage. Karnowski et al. (34) reported that OCT could visualize not only nuclei but also pronuclei and nucleoli in mouse early embryos and blastocysts. They also used OCT to show time dependent changes in these nuclear architectures (34). In cattle, TLC analysis has revealed that the time-dependent changes, such as the time of the first cleavage and the subsequent number of blastomeres, and the number of blastomeres at the onset of the lag-phase are useful for selecting embryos with a development potential (4, 12–14). Since we also visualized binuclear cells in a blastomere at the 2-cell stage, which is known as a negative indicator for development (43), OCT should also be useful for weeding out poor quality embryos. Previous reports also indicate that morphological indices also help to select high quality bovine embryos (4, 13, 43). Furthermore, the structure of bovine blastocyst has been precisely quantified in the present study. Together, the above findings suggest that detecting time-dependent structural changes of early-stage bovine embryo by OCT could improve evaluations at the blastocyst stage.

The present study reports the first normal deliveries of calves following the transfer of OCT-analyzed bovine embryos. The present conception rate (50%) and the birth rate (40%) following OCT are typical for ETs, indicating that OCT did not adversely affect ET. Although a PCA was unable to identify the parameters associated with pregnancy, TE-related parameters may be useful for evaluating bovine embryos. At present, OCT imaging should be useful for investigating the time-dependent changes of IVF embryos, and with further improvements, be useful for the selection of high-quality embryos for transfer.

## Data Availability Statement

The original contributions presented in the study are included in the article/Supplementary Material, further inquiries can be directed to the corresponding author/s.

## Ethics Statement

Ethical review and approval was not required for the animal study because the presented results were based on the bovine blastocyst images captured in culture conditions, so that the present experiments did not have any stress to animals. Animal handling and experimental procedures in farms were carried out following the Guidelines for Proper Conduct of Animal Experiments by Science Council of Japan (http://www.scj.go.jp/ja/info/kohyo/pdf/kohyo-20-k16-2e.pdf). Written informed consent was obtained from the owners for the participation of their animals in this study.

## Author Contributions

RN: conceptualization, resources, data curation, supervision, project administration, and writing—review and editing. YM: methodology, investigation, and writing—original draft preparation. RH, YK, MK, and KU: methodology, investigation, and writing—review and editing. MH and TO: writing—review and editing. All authors have read and agreed with the manuscript for publication.

## Conflict of Interest

RH, YK, and MK were employed by SCREEN Holdings Co., Ltd. The remaining authors declare that the research was conducted in the absence of any commercial or financial relationships that could be construed as a potential conflict of interest.
